# Timing and frequency of high temperature events bend the onset of behavioural thermoregulation in Atlantic salmon (*Salmo salar*)

**DOI:** 10.1093/conphys/coac079

**Published:** 2023-01-18

**Authors:** Antóin M O’Sullivan, Emily M Corey, Elise N Collet, Jani Helminen, R Allen Curry, Chris MacIntyre, Tommi Linnansaari

**Affiliations:** FOREM, University of New Brunswick, Fredericton, Fredericton, New Brunswick, NB E3B 5A3, Canada; Canadian Rivers Institute, University of New Brunswick, New Brunswick, NB E3B 5A3, Canada; O’Sullivan Ecohydraulics Inc., Fredericton, New Brunswick, Canada; Canadian Rivers Institute, University of New Brunswick, New Brunswick, NB E3B 5A3, Canada; Biology, University of New Brunswick, Fredericton, Canada; FOREM, University of New Brunswick, Fredericton, Fredericton, New Brunswick, NB E3B 5A3, Canada; Canadian Rivers Institute, University of New Brunswick, New Brunswick, NB E3B 5A3, Canada; Natural Resources Institute Finland, Helsinki, Uusimaa, 00790, Finland; FOREM, University of New Brunswick, Fredericton, Fredericton, New Brunswick, NB E3B 5A3, Canada; Canadian Rivers Institute, University of New Brunswick, New Brunswick, NB E3B 5A3, Canada; Biology, University of New Brunswick, Fredericton, Canada; FOREM, University of New Brunswick, Fredericton, Fredericton, New Brunswick, NB E3B 5A3, Canada; FOREM, University of New Brunswick, Fredericton, Fredericton, New Brunswick, NB E3B 5A3, Canada; Canadian Rivers Institute, University of New Brunswick, New Brunswick, NB E3B 5A3, Canada; Biology, University of New Brunswick, Fredericton, Canada

**Keywords:** underwater camera, thermal refuge, thermal hysteresis, thermal aggregation, salmonid, Atlantic salmon

## Abstract

The role of temperature on biological activities and the correspondent exponential relationship with temperature has been known for over a century. However, lacking to date is knowledge relating to (a) the recovery of ectotherms subjected to extreme temperatures in the wild, and (b) the effects repeated extreme temperatures have on the temperatures that induce behavioural thermoregulation (aggregations). We examined these questions by testing the hypothesis that thermal thresholds which initiate aggregations in juvenile Atlantic salmon (AS) (*Salmo salar*) are not static, but are temporally dynamic across a summer and follow a hysteresis loop. To test our hypothesis, we deployed custom-made underwater camera (UWC) systems in known AS thermal refuges to observe the timing of aggregation events in a natural system and used these data to develop and test models that predict the temperatures that induce thermal aggregations. Consistent with our hypothesis our UWC observations revealed a range of aggregation onset temperatures (AOT) ranging from 24.2°C to 27.1°C, thus confirming our hypothesis that AOTs are dynamic across summer. Our models suggest it take ~ 11 days of non-thermally taxing temperatures for the AOT to rebound in the study river. Conversely, we found that as the frequency of events increased, the AOT declined, from 27.1°C to 24.2°C. Integrating both model components led to more robust model performance. Further, when these models were tested against an independent data set from the same river, the results remained robust. Our findings illustrate the complexity underlying behavioural thermoregulation in AS—a complexity that most likely extends to other salmonids. The frequency of extreme heat events is predicted to increase, and this has the capacity to decrease AOT thresholds in AS, ultimately reducing their resilience to extreme temperature events.

## Teaser

Studies on the temporal aspects of *when* thermal refuges are used by stenothermic fishes remain limited. We found the temperatures that induce thermal refuge use by Atlantic salmon juveniles are dynamic across a summer. Our results illustrate that these temperatures can vary by #x007E;#x2009;2.8#x00B0;C within an 11-day window.

## Introduction

The influence of temperature on the functioning of biota is pervasive. Perhaps nowhere is the fingerprint of temperature more apparent than in the metabolic rates of biota (see Brown *et al.,* 2004; Clarke, 2006). The metabolic rate and mass influences the sustenance of all life, from unicellular to multicellular organisms. The dependence of metabolic rate on temperature influences the global distribution of coldwater stenotherms, such as Atlantic salmon (*Salmo salar*) (Elliott and Elliott, 2010; Morelli *et al.,* 2020). Whilst salmonids generally occupy cool rivers, extreme heat events can lead to ambient thermal regimes that exceed critical thermal tolerance thresholds (Elliot, 1991; Frechette *et al.,* 2021). The effects of exposure to critical thermal regimes are metabolically, physiologically and energetically costly (Lennox *et al.,* 2018; Little, Loughland, and Seebacher, 2020; Morash *et al.,* 2021). When temperatures exceed critical thresholds in natural settings, salmonids seek out cool-water thermal refuges to offset physiological and energetic stresses induced by the thermal conditions of the river (Corey *et al.,* 2020; Ebersole, Liss, and Frissell, 2003; Huntsman, 1942; Keefer and Caudill, 2016). This thermoregulatory behaviour is ubiquitous amongst salmonids from juveniles to adults and highlights the importance of cool-water refuges for the survival of salmonids (O’Sullivan *et al.,* 2021a; Torgersen *et al.,* 1999).

In ecology, the term kinetics is used to describe metabolic rate as a function of temperature (Brown *et al.,* 2004; Clarke, 2006). This relationship can be decanted into a simple exponential model, and has implications for all of Earth's biota, from ants (Shapley, 1924) and bovine (Parkhurst, 2010) to vegetation (Hollister, Webber, and Bay, 2005). Indeed, the role of temperature on biological activities and the correspondent exponential relationship with temperature has been known for over a century (Brown *et al.,* 2004—see Boltzmann, 1872; Arrhenius 1889). However, biological activities cannot increase exponentially in perpetuity; at some point, the organism must reduce its temperature, or die (Parkhurst, 2010; Corey, 2022); alas, behavioural thermoregulation. Myriad studies have given credence to these mechanistic understandings; for example, (Santos, Castañeda, and Rezende, 2011) used the Gompertz equation (Gompertz, 1825) to examine the heat tolerance in small fruit flies (*Drosophila*). However, lacking in literature (at least to the authors knowledge) are investigations establishing (a) the recovery of ectotherms subjected to extreme temperatures in the wild, and (b) the effects repeated exposure to extreme temperatures may have on ectothermic organisms.

Contemporary research has revealed species-specific and geographic variability in the water temperatures that induce behavioural thermoregulation in salmonids (Brewitt, Danner, and Moore, 2017; Corey *et al.,* 2020; Sutton, Deas, Tanaka, Soto, and Corum, 2007). Whilst some studies have found specific temperatures induce movements to thermal refuges (Dugdale *et al.,* 2016; Corey *et al.,* 2020), others have found a range of temperatures (Ebersole *et al.,* 2001; Sutton *et al.,* 2007). In rainbow trout, for instance, no consistent temperature was found to induce thermal refuge use; rather, the movements occurred over a range of temperatures between 18.0 and 25.7°C (Ebersole *et al.,* 2001). These findings differ from those observed for juvenile coho salmon (*Oncorhynchus kisutch*) where thermal refuge use started between 22.0 and 25.0°C (see Brewitt and Danner, 2014). Similarly, Wilbur *et al.* (2020) found juvenile Atlantic salmon used thermal refuges at temperatures between 25°C and 27°C. This leaves the question, what might explain this variability in aggregations onset temperatures?

The phenomenon of hysteresis is inherent in biotic and abiotic processes, and therefore has found widespread use in the fields of physics, hydrology and kinesiology, to mention but a few ((Jiles, 1994; Brassard *et al.,* 2017; Wondzell and Ward, 2022). Most simply, hysteresis can be summated as follows: the state of a system depends on what has happened to it in the past and what is happening to it in the present. With that, one can conceptualize that the effect of a past extreme temperature event on an ectotherm will influence the physiological condition of the organism in the present. This relationship can be also be used to conceptualize the effects of the repeated thermal stress on the thermal thresholds that induce behavioural thermoregulation in salmonids, or in this study, juvenile Atlantic salmon. Conceptually, by increasing the number of bahavioural thermoregulation events over a window of time, the temperature that induces such behaviour will decrease. Conversely, as the time since a behavioural thermoregulation event increases the fish would recover, thereby returning its thermal threshold to its upper limit.

In this study, we hypothesize that the thermal thresholds underpinning behavioural thermoregulation in juvenile Atlantic salmon are not static, but are temporally dynamic within a summer. To test our hypothesis, we developed and deployed custom-made underwater camera systems in known Atlantic salmon thermal refuges to observe the timing of behavioural thermoregulation events in a natural system. We used these data to develop and test a suite of new models to predict the timing of behavioural thermoregulation based on the theory of hysteresis; that is, timing of behavioural thermoregulation (modelled state) is inherently dependent on the “history” of previous thermoregulation events of the exposed individuals, resulting in variable, rather than static, threshold temperature.

## Methods

### Study area

**Figure 1:**
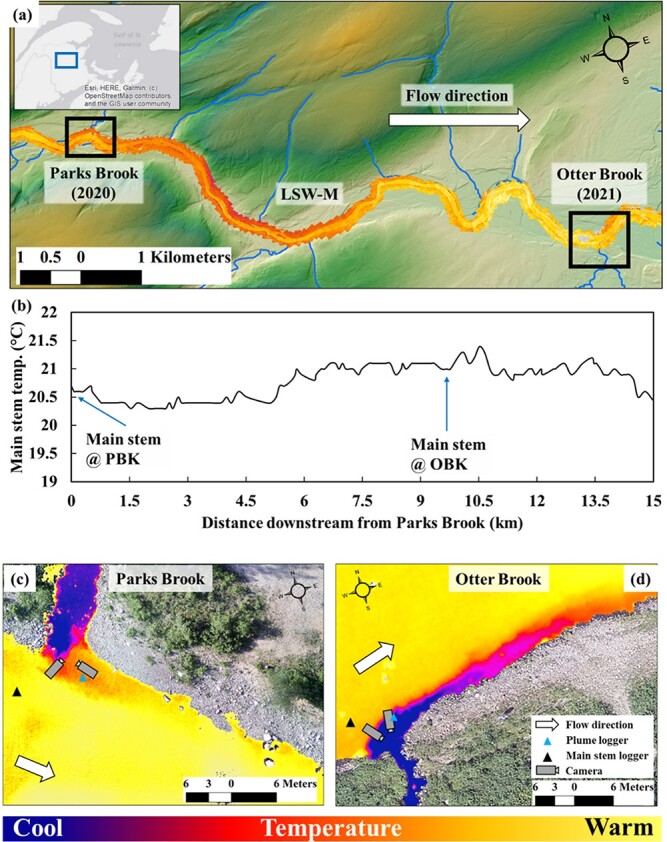
A map showing the study sites (Parks Brook (2020 data) and Otter Brook (2021 data)) superimposed on a thermal infrared image (TIR) of the study river, the Little Southwest Miramichi, New Brunswick, Canada (LSW-M; Panel a). The thermal profile between Parks and Otter Brook is presented in (b) while (c) and (d) delineate the Park and Otter Brook thermal refuges, respectively; and include the camera orientation within refuges and plumes and location of main-stem temperature loggers.

This study was conducted in the Little Southwest Miramichi (LSW-M) river, a tributary of the Miramichi River, New Brunswick, Eastern Canada—Figure 1a. The LSW-M has a topographic drainage area ~ 1300 km^2^ and is climatically characterized by cold winters and warm summers (Caissie, Breau, Hayward, and Cameron, 2013; Linnansaari and Cunjak, 2010). In summer, maximum water temperature across the Miramichi catchment displays a wide range of variability, with some tributaries measuring > 30°C and others ~ 15.0°C (O’Sullivan *et al.,* 2021b). The Miramichi region was once the top producer of Atlantic salmon in North America; however, the population is in a state of steep decline since at least the 1970s (Samways, 2017).

Our study sites are two known thermal refuges on the LSW-M. The upstream thermal refuge is Parks Brook (Figures 1a, b). Parks Brook has a topographic drainage area ~ 19 km^2^, and is groundwater influenced (O’Sullivan, Linnansaari, and Curry, 2019; O’Sullivan *et al.,* 2021b—Figure 1b). The second thermal refuge, Otter Brook, is located ~ 8.5 km downstream of Parks Brook, and has a topographic drainage area ~ 11 km^2^ (Figure 1a). Otter Brook is more groundwater dominated than Parks Brook (Kurylyk *et al.,* 2014; Morgan and O’Sullivan, 2022; O’Sullivan, Linnansaari, and Curry, 2019), and is relatively cooler with a substantially longer thermal plume (or thermal effect) than Parks Brooks (Figure 1b and c).

Thermographs (Hobo UA-002-64 Pendant Temperature/Light data logger—64 KB) housed in a white uPVC pipe were mounted to cinder blocks and subsequently stationed in the two thermal refuges. Thermographs were placed in the main stem LSW-M, slightly upriver and adjacent to Parks Brook and Otter Brook thermal plumes, and within each thermal plume (Figure 1b and c). Temperatures were recorded every 30 minutes between 16 June and 31 August 2020 at the Parks Brook refuge and between 1 June and 31 August 2021 at the Otter Brook refuge.

### Main river and refuge thermal regimes

To establish if the thermal regimes of the main river and hydrogeologically distinct thermal refuges (Kurylyk, Bourque, and MacQuarrie, 2013; O’Sullivan *et al.,* 2021b) differed we compared the regimes within and across years. As the maximum temperature is the most critical metric to drive the onset of thermal aggregations, we compared the daily maximum thermal regimes. We compared (a) the main river and thermal refuge temperature within a summer, i.e. main river compared with thermal refuge, (b) the main river summer temperatures between years, i.e. 2020 compared with 2021, and (c) the thermal refuges between years, i.e. 2020 compared with 2021. As the sample sizes differed between years, we performed a series of Mann–Whitney U tests. Further, in 2020 the Parks Brook thermal refuge temperature logger was highly influenced by thermal mixing with the main river until 24 June, 2020, at which point the logger was moved further into the plume. As these data were excluded from statistical comparisons, this created an uneven and non-normally distributed sample set to compare the within years difference for the 2020 data set, and further supporting the necessity to use non-parametric analyses. In all tests α = 0.05.

### Underwater cameras

#### Custom-made video cameras—2020

**Figure 2:**
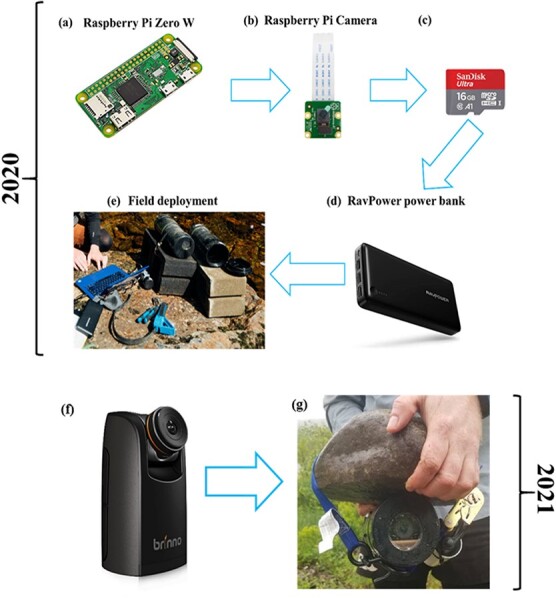
A schematic detailing the components of the custom-made underwater camera for 2020 (the Raspberry Pi system) and 2021 (the Brinno system). The Raspberry Pi systems are video cameras that collected a 30 second video every hour from 05:00 to 21:00 each day of the studied. The Brinno system is a time-lapse camera that collected a photo every 10 minutes from 05:00 to 21:00 each day of the study. A full description of these systems is provided in the main text.

A custom-made underwater camera system was developed by coupling a Raspberry Pi Zero W—microcomputer with a Raspberry Pi Camera Module V2–8 Megapixel, 1080p, (red-green-blue) RGB spectra (RPI-CAM-V2—Figure 2a and b). The system was programmed to turn on at the top of each hour between 05:00 and 21:00. During this time, the system recorded a video for 30 seconds, after which it was commanded to shut off, and all data was saved to a 16-GB micro SD card (Figure 2c). This command structure maximized the power bank charge (Portable Charger RAVPower 26800mAh Power Bank 26800—Figure 2d). The camera system was housed inside a custom-made acrylonitrile butadiene styrene (ABS) pipe, diameter = 40 mm, with covers on both ends. This camera housing was placed, together with the power bank and a desiccant (*i.e.* silica gel beads), into a larger ABS pipe (diameter = 100 mm), with a transparent acrylic sheeting lens glued to the permanent lid of the ABS pipe; a threaded lid was attached to the opposite end thus providing an access point (Figure 2e). All ABS joints were fused with ABS adhesive and additional waterproofing silicon was applied. The ABS housing was mounted to a cinder block using a steel wire (Figure 2e). Two underwater cameras were deployed at the Parks Brook refuge between June 16 and August 31, 2020, and orientated as illustrated in Figure 1b. When river temperatures were < 27°C until June Finally, the site was visited every 7 days to download data and to change the power bank.

#### Time-lapse still cameras—2021

For the summer of 2021, we sought to increase the temporal resolution of our cameras, whilst also increasing the battery life of the system. To do this, we used Brinno TLC200 Pro Time-Lapse Cameras © (Figure 2f). These cameras have an image resolution of 720p, and dependent upon temperature and shooting interval, the four AA batteries can last up to 42 days. The Brinno cameras were placed in a custom-made housing identical to the 2020 design (Figure 2g). The cameras were programmed to take still photos every 10-minutes from 05:00 to 21:00, between May 31 and September 2 2021. Two camera units were deployed at the Otter Brook refuge, and their orientation is illustrated in Figure 1c.

### Definition of aggregation observations

**Figure 3:**
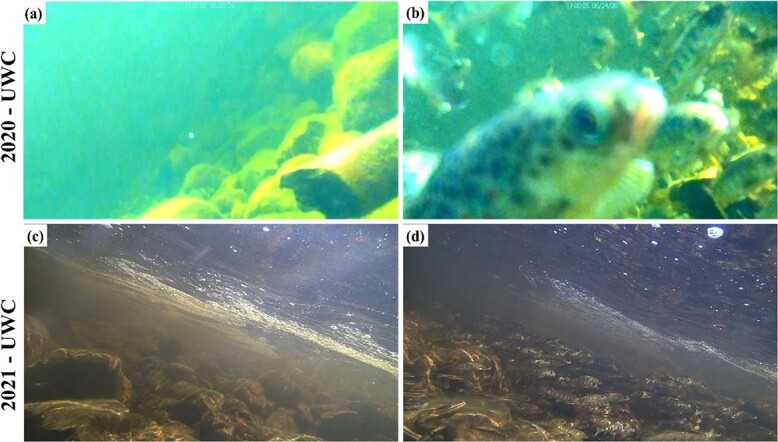
An example of underwater camera photos captured with each system. Panels (a) and (b) are images from the Raspberry Pi camera system in the Parks Brook refuge when it is void of fish during non-stressful temperatures, and when juvenile Atlantic salmon are using the refuge during thermally stressful conditions, respectively. Images from the Brinno TLC200 Pro Time-Lapse Cameras © show the Otter Brook refuge during 2021 void of fish during non-stressful temperatures (c), and when juvenile Atlantic salmon, and other species (Brook trout and black nose dace), using the refuge during thermally stressful conditions (d).

The role of underwater cameras in both years was to collect date-time information on the timing of behavioural thermoregulation aggregation events by juvenile Atlantic salmon. For the purposes of this study the onset of a behavioural thermoregulation event was defined as the presence of ≥10 Atlantic salmon parr (Corey *et al.,* 2020; Dugdale *et al.,* 2016; Figure 3). In some instances, aggregations can remain in place for days (*e.g.*Corey, 2022). As the focus of this study was the onset temperature of thermal aggregations, we defined the onset of a new aggregation as one where the prior aggregation had dispersed. These events were easily separated from the baseline non-aggregation events due to the general low density of juvenile Atlantic salmon in the Miramichi River (Chaput, Douglas, and Hayward, 2016) and their territorial nature during non-thermal events ((Linnansaari and Cunjak, 2010) (Figure 3). The high resolution of our underwater camera videos and images allowed confident identification of aggregating fishes to species (i.e. juvenile Atlantic salmon); the only other coldwater stenothermic salmonid in the studied area is brook trout (Figure 3). Whilst brook trout (both juvenile and adult) were also commonly observed in our imaging, their density in the studied area, and therefore frequency in our imaging, was very low. Furthermore, brook trout were generally easily identifiable due to the size differences (see *e.g.*Figure 3d for an adult brook trout within an aggregation), or due to their white leading edge in their anal fin, and the lack of easily identifiable “parr marks” typical for juvenile Atlantic salmon. Additionally, some blacknose dace (*Rhinichthys atratulus*) were observed in our imagery; however, these were also easily identifiable by the markings.

Corey (2022) found once an aggregation event has occurred within the LSW-M, juvenile salmon display high fidelity towards reaches with the thermal refuges. In such instances, the juvenile salmon abandoned the reach they were located in prior to the aggregation event, if the reach did not contain a thermal refuge. This fidelity towards reaches with the thermal refuges remained until the autumn, when fish returned to abandoned reaches. Coupling the similar thermal regimes between our study sites, and the findings on refuge fidelity and abandonment of territories without refuges (as per Corey *et al.* [in review]), we make the assumption that the majority of the fish we observed are consistently using the refuges.

### Analytical models

Previous empirical *in situ* investigations in Atlantic Canada and affiliated physiological experiments to better understand behavioural thermoregulation in juvenile Atlantic salmon have found sigmoidal shaped responses to thermal stress (Breau *et al.,* 2011; Corey, Linnansaari, Cunjak, and Currie, 2017; Corey, 2022). From these studies, we conceptualized that the sigmoidal curves represent half of a hysteresis loop. As such, we deduced that to model aggregation onset temperatures (hereafter AOT), two model components will be required. We included two mathematical components that relate the time since physiologically challenging conditions have been observed (Time since Event [TsE] and their frequency (Frequency of Events [FoE]) to model AOT.

**Figure 4:**
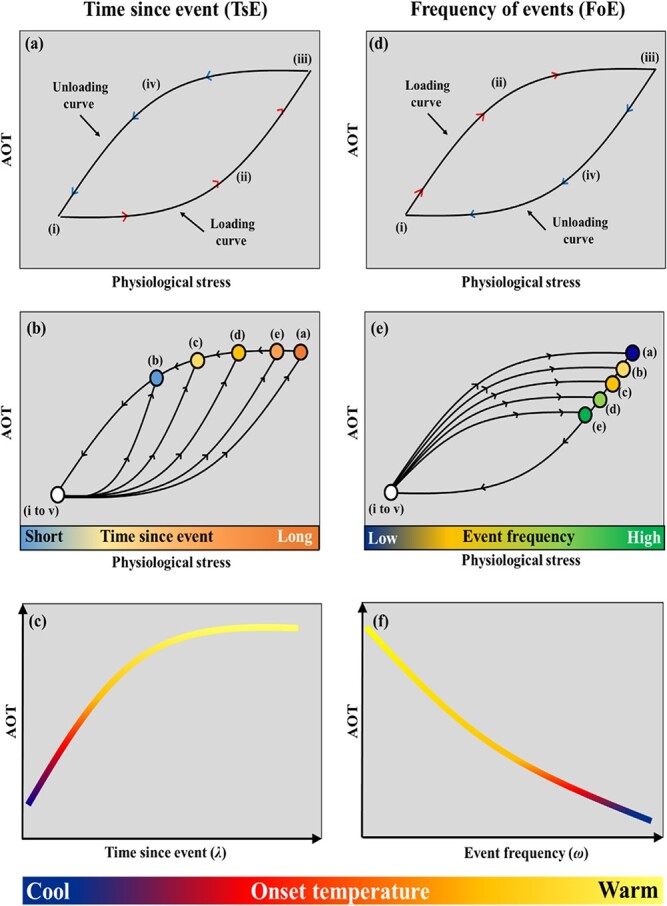
The development of our conceptual hysteresis-based models. Panels a-c details the development of the time since event (TsE) model, and Panels d-f details the development of the frequency of events (FoE) model. In panels (a and d) the fish is conceptualized to begin to accumulate physiological stress at point (i), with stress increasing along the loading curve (ii) until a point of stress saturation is reached (iii). At this point, the fish will seek thermal refuge and defines the aggregation onset temperature (AOT). Once in the refuge, the stress reduces along the loading (iv). A full and detailed description of these models is provided in the main text. Panels (b and e) illustrate how the TsE and FoE models vary through time as a function of time since an event and event frequency, respectively. Finally, panels (c) and (f) illustrate each model as single line.

For the TsE component, the conceptual relationship between AOTs and physiological stress for juvenile Atlantic salmon is shown via a loading curve, where (i) details the inflection point of accumulating physiological stress (induced by temperature) (Figure 4a). As temperature increases, physiological stress accumulates exponentially (ii), as has been empirically demonstrated in laboratory studies (Cindy Breau *et al.,* 2011; Corey *et al.,* 2017). However, the exponential increase in physiological stress cannot continue in perpetuity; the fish have a critical thermal maxima dictated by their physiological constraints (Corey *et al.,* 2017; Morash *et al.,* 2021). We conceptualize that when a point of physiological stress saturation is reached, a thermal refuge will be sought by juvenile salmon in nature (iii—Figure 4a; *i.e.* AOT). Once a thermal refuge is found, the temperature begins to reduce, consequently alleviating physiological stress. As the loading curve is exponential, we conceptualize the unloading curve will be the inverse, or mathematically, the unloading curve will follow a logarithmic function. These curves intercept and thus close the system, representing a loop (Figure 4a).

Under our conceptual model, the subsequent points of AOT are driven by prior physiological stress history, and are thereby represented by the concept of thermal hysteresis (Figure 4b). With that, point (i) illustrates a hypothetical inflection point where physiological stress begins to accumulate and leads to the initial aggregation defined at point (a). In this instance, the fish’s physiological stress threshold is lower as it has not had sufficient time for full metabolic recovery; it will therefore have a lower thermal aggregation threshold (point b; Figure 4b). As time since the aggregation event increases, the AOT thresholds increases—points c, d, e, respectively (Figure 4b), until a full metabolic recovery is achieved, and the hysteresis loop is reset. In the hysteresis loop, whilst the inflection points—points ii, iii, iv, and v are considered to be static—the fish’s lower physiological stress threshold leads to shorter pathway(s) to AOT, in comparison to a fully reset baseline condition. Mathematically, the TsE component of the AOT model takes the form:

(1) \begin{equation*} TsE=a\times \log \lambda +b \end{equation*}

where *TsE* is the aggregation onset temperature, *a*, and *b* are empirically derived coefficients, and *λ* is the time since an event. In this study, we measured λ in days. The units for *a* and *b* are in temperature (°C or F).

A simplified illustration of the TsE model component is presented in Figure 4c. The AOT as a function of time since event is shown as a logarithmic curve, and is positively related to onset aggregation temperature.

In addition to the TsE component (time required for full metabolic recovery), we conceptualized that the frequency of aggregation events (FoE) can reduce the fish’s thermal threshold. We postulate that this will also take the form of a hysteresis loop; however, the FoE loop’s loading/unloading is the inverse of the TsE loop (Figure 4a, d). We conceptualize that the unloading curve for the FoE model component will be an exponent function (Figure 4d). Similar to the TsE component, point (i) is the inflection point at which the physiological stress is initiated and increases as a function of temperature (Figure 4d). This increase follows a logarithmic trend (ii) until a physiological stress saturation point (*i.e.* AOT) is reached (iii) (Figure 4d). At this point, thermal refuge is sought, and the unloading curve follows an exponential trend (iv—Figure 4d), thereby completing a loop.

Similar to the TsE model, the AOT points are fluid for the FoE model, and the process is governed by a hysteresis loop where increasing frequency of events reduces the AOT (Figure 4e). Increasing the event frequency by one event only moderately reduces the AOT to (b). As the event frequency increases, the thermal aggregation points decrease—points c, d, e, respectively (Figure 4e). This contraction characterizes a decrease in the fish’s thermal threshold. Mathematically, this FoE model component takes the form:

(2) \begin{equation*} FoE=c{e}^{d\omega} \end{equation*}

where *c* and *d* are empirically derived coefficients, and *ω* is the reduction rate. All units are in temperature (°C or F). The *c* parameter denotes the temperature of the first aggregation threshold, or events after full recovery has been achieved and has units of temperature (°C or F). The *ω* parameter takes the form:

(3) \begin{equation*} \omega =\frac{\sigma }{T} \end{equation*}

where *σ* is the number of events over a period (*T*). *σ* and *T* are empirically derived from the underwater camera observations. Finally, equation (3) is substituted in to equation (2), giving:

(4) \begin{equation*} FoE=c{e}^{d\frac{\sigma }{T}} \end{equation*}

We used a sensitivity test to establish the best fit *T* across the aggregation onset observations. This was completed using a moving window, where the window was defined by the frequency of unique aggregation events within *T* values ranging from 7 to 14 days (Mesa, Weiland, and Wanger, 2002).

A simplified illustration of the FoE model component is presented in Figure 4f. The onset aggregation temperature as a function of event frequency is shown as an exponential curve, and this is negatively related to the onset aggregation temperature.

Both model components are necessary to predict the temperature at which juvenile Atlantic salmon aggregate in thermal refuges. To account for the inherent interactions between the time since a previous aggregation (TsE) and aggregation frequency (FoE), we developed an integrate model. Mathematically, this final model takes the form:

(5) \begin{equation*} {T}_{integrated}=a\times \log \lambda +b+c{e}^{d\frac{\sigma }{T}} \end{equation*}

where the model parameters are detailed in equations (1), (2), (3), and (4) above. Similarly, all units are in temperature (°C or F).

In each year, *n—*1 data points were used to develop and test the models. This was required as the first aggregation provides a baseline from which to calculate time since an aggregation and the frequency of aggregations for the next sequential aggregation. The 2021 data set was used to develop the model coefficients, and the 2020 data set was used to independently test the models. In each year, the main stem temperature loggers in tandem with the underwater camera observation were used to quantify the temperatures that induced the onset of behavioural thermoregulation. All of the models were developed in MATLAB © using the curve fitting application. Model performance was based on the coefficient of determination (R*^2^*), sum of squared errors (SSE) and root mean square error (RMSE). The above metrics and Akaike information criterion (AIC) were also used for the sensitivity test to establish the best fit *T* equation (5).

## Results

### Thermal regimes and aggregation observations

**Figure 5:**
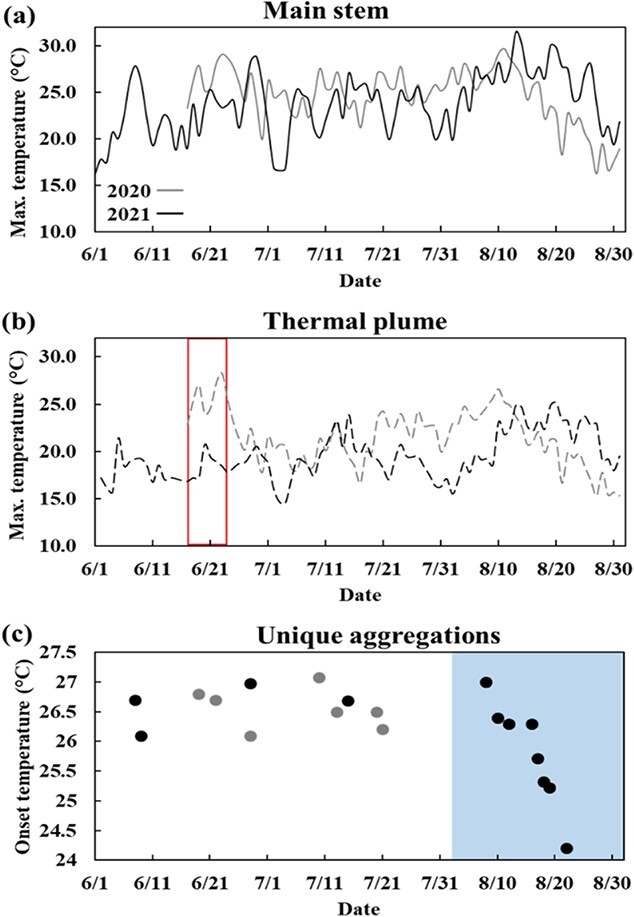
The main stem maximum water temperatures observed on the LSW-M during the summer of 2020 (grey line) and 2021 (black line) is shown in (a). The corresponding temperature of the thermal plumes is shown in (b), where the grey dashed line relates to Parks Brook and the black dashed line is Otter Brook. Between June 17 and 24 in 2020, the Parks Brook temperature logger was influenced by thermal mixing from the main stem and was subsequently moved further into the plume; the affected time period is shown in red. The onset temperature of thermal aggregations is shown in (c) where the grey and black dots reflect 2020 and 2021 observations, respectively. Of note is the blue polygon which delimits a time-period during 2020 when the cameras malfunctioned, rendering the omission of data from this period—see text.

The thermal regime of the LSW-M during summer of 2020 was characterized by an average, maximum and minimum water temperature of 21.8, 29.7, 12.0°C, respectively, with a S.D. of 3.3°C (Table 1; Figure 5a). The thermal plume of Parks Brook in 2020 had an average, maximum and minimum temperature of 18.8, 26.6, 11.6°C, respectively, with a S.D. of 2.8°C (Table 1). During summer of 2021 the LSW-M average water temperature was 1°C cooler (20.8°C) than in 2020, whilst the maximum was 1.8°C warmer (31.5°C), and the minimum was 0.7°C cooler (11.3°C), with a S.D. of 3.5°C (Table 1). The thermal plume of Otter Brook during 2021 had an average, maximum and minimum temperature of 17.8, 25.1, 10.7°C, respectively, with a S.D. of 2.3°C. A complete time-series of daily maximum water temperatures in each year is shown in Figures 5a and b. Both thermal refuges had maximum daily temperatures that were significantly cooler than their corresponding main river temperatures in each studied year (Table 2).

**Table 1:** A summary of main river (T_r_), thermal refuge (T_pl_) and thermal aggregation onset temperatures (T_on_) for 2020 and 2021

**Statistic**	**2020**			**2021**		
	T_r_ (°C) (*n* = 3648)	T_pl_ (°C) (*n* = 3264)	T_on_ (°C) (*n* = 7)	T_r_ (°C) (*n* = 4416)	T_pl_ (°C) (*n* = 4332)	T_on_ (°C) (*n* = 12)
Mean	21.8	18.8	26.5	20.8	17.8	26.2
Maximum	29.7	26.6	27.1	31.5	25.1	27.0
Minimum	12.0	11.6	26.1	11.3	10.7	24.2
SD	3.3	2.8	0.4	3.5	2.3	0.8

The 2020 and 2021 data is from Parks Brook and Otter Brook, respectively.

**Table 2:** Statistical analyses results comparing main river and thermal refuge thermal regimes across sites and years, where T_r_ is the main river temperature and P_l_ is the thermal plume temperature

Comparison	*U*	*U* (standardized)	Expected value	Variance (*U*)	*p*-value (two-tailed)
2020 main river and thermal refuge max. Daily temperatures *n*_(Tr)_ = 76; *n*_(Pl)_ = 69	928	−6.7	2622.0	63787.6	**<0.0001**
2021 main river and thermal refuge max. Daily temperatures *n*_(Tr)_ = 92; *n*_(Pl)_ = 91	7075	8.1	4186.0	128348.9	**<0.0001**
2020 and 2021 main river max. Daily temperatures *n*_(2020)_ = 76; *n*_(2021)_ = 92	2881.5	−2.0	3496.0	98446.6	0.05
2020 and 2021 thermal refuge max. Daily temperatures *n*_(2020)_ = 69; *n*_(2021)_ = 91	4261.5	3.9	3139.5	84219.0	**<0.001**

The bolded values denote those that are statistically significant.

During 2020, seven unique thermal aggregations were observed during the period of camera operation (Figure 5c). The first aggregation in 2020 occurred June 19^th^ (Figure 5c). Beginning on 27 July 2020, a camera malfunction occurred (see Figure 5c). This malfunction led to the cameras turning on and off randomly, and upon inspection of the camera components and source code, no cause was found. We did observe aggregations during this time (27 July to 14 August 2020); however, the gaps in the data prevented the use of these observations as there was uncertainty around the timing of aggregation onset. The average AOT during the operational period of the cameras for 2020 was 26.5°C, with a maximum, minimum, and S.D. of 27.1°C, 26.1°C and 0.4°C, respectively (Table 1).

During 2021, the issues that occurred during 2020 were remedied by using the high temporal resolution Brinno © camera system. Twelve unique thermal aggregations occurred in 2021, with the earliest occurring on 8 June, and the highest frequency of events occurring during August (Figure 5c). The average AOT during the operational period for 2021 was 26.2°C, with a maximum, minimum and S.D. of 27.0, 24.2 and 0.6°C, respectively (Table 1).

### Analytical model results

**Figure 6:**
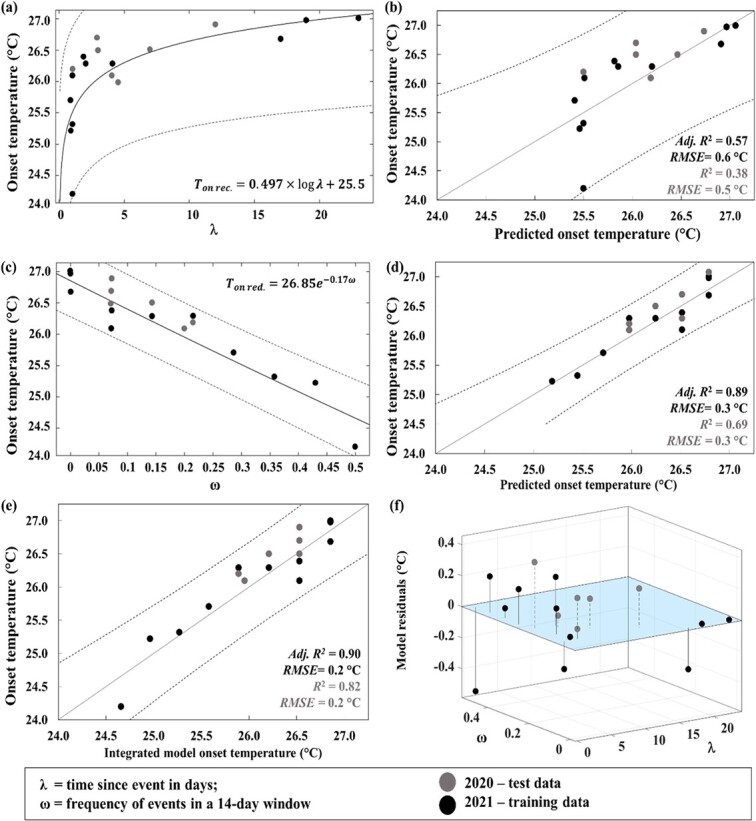
Panels (a) and (b) are graphical illustration of the time since event model (TsE) relative to 2020 (grey dots) and 2021 (black dots) observations, and the correspondent model performances. Similarly, panels (c) and (d) detail the frequency of events model (FoE) relative to 2020 and 2021. The integrated model results are shown in (e) and (f) highlights the model residuals.

A total of *n* = 11 unique aggregation onsets from the 2021 data were used to calibrate the TsE model, and the model coefficients are provided in Table 3. The resulting model had an *Adj. R^2^* = 0.49, a *SSE* = 2.8°C, and a *RMSE* = 0.61°C (Table 3; Figure 6a and b). Testing the TsE model against 2020 data produced an *R^2^* = 0.38; *SSE* = 1.18°C and a *RMSE* = 0.54°C (Table 3; Figure 6b).

**Table 3:** Summaries for all models; the coefficient symbols relate to equations (1), (4) and (5)

Model	Coefficients	Training *R*^2^	Adj. *R*^2^	Training SSE	Training RMSE	Test *R*^2^	Test SSE	Test RMSE
Time since Event (TsE)	a = 0.497	0.61	0.57	2.80	0.56	0.38	1.18	0.54
	b = 25.5							
Frequency of Events (FoE)	c = 26.85	0.90	0.89	0.74	0.29	0.69	0.37	0.30
	d = −0.17							
Integrated model	a = 0.15	0.91	0.90	0.64	0.23	0.82	0.37	0.22
	b = −1038							
	c = 1065							
	d = −0.003							

The model coefficients are derived from the 2021 data set, and the independent test data correspond to the 2020 data set

A suite of sensitivity models were used for the FoE model and a 14-day window was selected best time window to examine the role of event frequency as it had the lowest AICc = −20.7 value (Table 4). Using the 14-day window, the resulting FoE model had an *Adj. R^2^* = 0.89, a *SSE* = 0.64°C, and a *RMSE* = 0.29°C (Table 3; Figure 6a and b). Testing the FoE model component against 2020 data produced an *R^2^* = 0.69; *SSE* = 0.37°C and a *RMSE* = 0.30°C.

**Table 4:** The results of the range of moving windows used to select the best model fit for the thermal reduction model equations (2) to (4)—see text

Frequency sensitivity models (*n* = 11)									
Window	Model components	Coefficients	*R^2^*	SSE	RMSE	AIC_c_	∆AIC_c_	*w_i_*	Evidence ratio
14	c	26.85	0.90	0.74	0.29	−20.7	0	0.77	1.00
	d	−0.17							
13	c	26.85	0.85	1.09	0.35	−16.4	4.31	0.09	8.62
	d	−0.16							
12	c	26.85	0.85	1.09	0.35	−16.4	4.31	0.09	8.62
	d	−0.15							
11	c	26.83	0.77	1.62	0.43	−12.0	8.66	0.01	76.11
	d	−0.14							
10	c	26.82	0.70	2.17	0.49	−8.8	11.88	0.00	379.08
	d	−0.14							
9	c	26.84	0.71	2.09	0.23	−9.3	11.42	0.00	301.14
	d	−0.13							
8	c	26.91	0.76	1.74	0.44	−11.3	9.41	0.01	110.53
	d	−0.14							
7	c	26.94	0.81	1.33	0.38	−14.3	6.46	0.03	25.26
	d	−0.15							

The integrated model, which accounts for both TsE and FoE components, produced an *Adj. R^2^* = 0.90, a *SSE* = 0.74°C, and a *RMSE* = 0.23°C (Table 3; Figure 6e). Testing the integrated model against 2020 data produced an *R^2^* = 0.82; *SSE* = 0.37°C and a *RMSE* = 0.22°C (Table 3). The model residuals and relationships between each model parameter and thermal thresholds are shown in Figure 6f.

## Discussion

### Underwater cameras for behavioural thermoregulation studies

In freshwater ecology/biology, tagging biota has provided multitudinous insights into movements, drivers of movements, life history strategies and habitat use, to mention a few (Andrews *et al.,* 2020; Curry, Bernatchez, Whoriskey, and Audet, 2010). However, some research suggests long-term risks associated with tagging, such as tissue infections (*e.g.* Adams *et al.,* 1997). Such risks are particularly problematic when studying at risk species, such as the declining Miramichi Atlantic salmon population. Our goal was to develop a method that is passive (thus, non-invasive), low-cost and can operate independent of an external power source, *i.e.* remote regions. The underwater camera systems method met all these criteria: (a) the optical sensor facilitated observations that were passive, *i.e.* we did not touch or disturb any fishes. We observed hundreds of Atlantic salmon parr, along with other species, such as brook trout (*Salvelinus fontinalis*) and white suckers (*Catostomus commersonii*); (b) The Camera systems are low-cost ~ $160–200, and are easy to construct and operate; and (c) the cameras were dependent on battery packs remove the need for an external power source. It is clear that this method is a highly efficient tool in the field of aquatic ecology, and has myriad applications. A major advantage of our underwater camera method compared with traditional tagging studies is it does not rely on a previously sampled subpopulation, but can assess the responses of any individual in the population responding to the stressor. A second major advantage is the utility of our systems for work in remote locations, where no power sources exist, such as arctic areas (Huusko *et al.,* 2007). Additionally, battery life could be extended by setting the cameras to collect data less frequently, thereby reducing the need for repeated site visits. While limitations are inherent due to the passive nature of the optical sensor applied in this study, future studies could integrate infrared sensors (IR) and IR—light-emitting diode (LED) lamps (Hermann, Chladek, and Stepputtis, 2020). A unit that combines optical and IR sensors, and LED lights would allow 24/7 collection of data, thus give complete temporal coverage.

### Thermal hysteresis

Our cameras revealed a relatively narrow thermal threshold for the first behavioural thermoregulation aggregation in Little Southwest Miramichi that was ~ 26.7°C to 27.1°C. This aligns to observations by Corey *et al.,* (2020) whom observed aggregation onset at ~ 27°C, and Lund, Caissie, Cunjak, Vijayan, and Tufts, (2002) whom observed heat shock protein (Hsp) 70 levels increased significantly at 27°C in the same river. However, for the overall data set, we observed ~ 2.8°C variance in these thermal aggregation onset thresholds. What explains this variability in thermal aggregation onset temperatures in this and many other previous studies?

We believe that the variability documented in AOTs is a matter of fish’s thermal history—thermal hysteresis—wherein the onset temperature to the *first* aggregation of the season (or to a subsequent thermal event but only after full metabolic recovery from any previous event) is a somewhat rigid population-specific threshold (see above). However, there is a marked reduction in the aggregation onset temperatures during *subsequent* thermal events. We propose this is likely caused by the latency in physiological stress metabolites (i.e. physiological thermal “baggage”) in fish's bodies; resulting in the necessity to aggregate in lower temperatures—candidly termed thermal hysteria. This thermal hysteria is not always the same in absolute numbers, however, it appears to be governed by a process that can be accurately modeled using time since previous thermally taxing events and their frequency as variables.

It is evident from our model results that the time since the onset of a previous aggregation event (TsE) plays a role in the variance of onset temperatures for thermal aggregations. Relatively few studies have examined the performance and recovery of salmonids after acute heat stress events and those that have are confined to the laboratory (see Gallant *et al.,* 2017; Lewis *et al.,* 2010; Lund and Tufts, 2003). Our results suggest that juvenile Atlantic salmon thermal thresholds do not return to pre-event thresholds until ~ 12 to 18 days after acute heat stress events in the LSW-M. Further, it is apparent that the recovery process initially occurs at an exponential rate and then plateaus towards the upper thermal threshold. A study on juvenile Chinook salmon (*Oncorhynchus tshawytscha*) established that acute thermal stress induced a 25-fold increase in liver Hsp 70, compared with a control group, and the metabolite presence lasted 2 weeks (Mesa *et al.,* 2002). Whilst it is not possible to ascertain the physiological drivers from our data, it may be that our TsE model component is mapping these physiological processes via flux in thermal thresholds. It is also evident that the TsE model’s performance decreased when tested against 2020 data. However, the 2020 data is from the Parks Brook thermal refuge, which is warmer than the Otter Brook thermal refuge. Further the thermal regime of the main river also differed between years. We propose two possible reasons for the reduction in our model’s performance: (a) fish that use the cooler Otter Brook refuge spend less time in thermally stressful conditions and therefore recovery occurs quicker, and this would most likely change the model coefficients between years, and (2) the main river thermal regime differs between years; this would likely change both metabolic and physiological processes, and the stress accumulation of the juvenile salmon.

A common concern in studies that have looked for changes in performance with acute thermal stress is that they typically only focus on a single acute heat shock event (*e.g.*Tunnah, Currie, and MacCormack, 2017; Gallant *et al.,* 2017). Mesa *et al.* (2002) brought this concern to light, and suggested that multiple, cumulative stressor situations are far more likely for the fish in the wild. Our camera observations and modeled outcomes offer credence to these concerns. The FoE model component had a relatively high predictive capacity for both the training and test data sets. This supports the notion of an accumulated effect, where an increase in the frequency of aggregations leads to a concomitant decrease in thermal aggregation onset thresholds. Moreover, at its upper limit this frequency increase can reduce thermal aggregation onset thresholds by ~ 2.8°C in an 11-day window. By integrating the conceptual mechanisms of both the TsE and FoE models, the integrated model’s predictive capacity increased, and its error decreased. These results illustrate the complexity underlying behavioural thermoregulation in Atlantic salmon—a complexity that most likely extends to other salmonids.

As the climate warms, the frequency of extreme heat events is predicted to increase (Brodeur *et al.,* 2015). This frequency increase has the capacity to decrease thermal thresholds for behavioural thermoregulation in salmonids, ultimately increasing the number of days required to offset an aggregation event/behavioural thermoregulation migration. With that, we encourage other researchers to test our hysteresis model in other rivers and on other species, both salmonid and non-salmonid. Whilst there are myriad factors that confound the development of a universal theory of aggregation thresholds for all ectotherms, we conceptualize an emergent mechanism may exist, similar to other thermal ecology theories (see Brown *et al.,* 2004).

### Management and conservation implications

The current paradigm when conducting research and implementing management strategies is to use species-specific thermal thresholds for salmonids, with these thresholds assumedly static through time. Isaak *et al.,* (2015) used a size threshold to delineate thermal habitats of bull trout (*Salvelinus confluentus*) and cutthroat trout (*Oncorhynchus clarkii*), whilst O’Sullivan *et al.* (2021b) used homogeneous thermal threshold criteria for Atlantic salmon and brook trout based on age class. In eastern Canada, Fisheries and Oceans apply a homogeneous thermal threshold to protect Atlantic salmon from angling during periods of heat stress (DFO, 2012). Our results reveal these true physiological thresholds are not static, rather they are dynamic and vary with exposure and time, and support a growing repertoire of research that highlights the deficiencies in using binary thresholds (Martin *et al.,* 2020; Fitzgerald and Martin, 2022). Our results have broad implications for our understanding of how salmonids are affected by extreme heat events, and how to design ecologically relevant management plans. One such suggestion is the development of real-time, river specific, thermal stress indices (TSI). For instance, our model could be coupled with real-time in-stream temperature data to provide a TSI. The TSI would vary as a function of temperature, thereby accounting for thermal stress threshold variance. Fisheries managers could the use this TSI tool to apply warm water closures that are ecologically meaningful. Such a tool would also be useful for scientists. Currently, electrofishing in New Brunswicks’ salmon rivers ceases when water temperatures > 23°C (DFO, 2013). This management protocol is designed to limit fish stress; however, our results indicate in the lower LSW-M, aggregation thresholds can be as low as 24.2°C. Breau *et al.,* (2011) found the amount of 2+ juvenile salmon displaying stress with increasing water temperature increased in a sigmoidal fashion (see Figure 2 in Breau *et al.,* 2011). The inflection point showing an increase in stress response occurred between 20–22°C and plateaued ~ > 26°C. Considering our results reveal these thermal thresholds can reduce, we suggest a dynamic water warm electrofishing protocol; one that reflects the variance in river thermal regimes and the concomitant time dependent response in juvenile salmon. This suggestion addresses the concerns posed by Corey *et al.,* (2017).

### Study limitations

We acknowledge there are inherent limitations in our study. It is possible that despite our best efforts, the UWCs did not capture all the fish that are aggregating within the thermal refuge due to the their placement and orientation. This could be remedied by placing more UWCs in the refuge to ensure that as much of the refuge is photographed as possible; thus, limiting the likelihood of missing data points. The decision to define an aggregation as the presence of ≥10 parr is arbitrary, but is based on previous studies and knowledge of parr density in this section of river (*e.g.* Breau, Cunjak, and Bremset, 2007; Dugdale *et al.,* 2016; Corey *et al.,* 2020). In the LSW-M, surveys conducted by DFO (2022) suggest the density of juvenile salmon is stable with > 35 fish 100 m^−2^; however, in rivers with lower densities the definition of what classifies an aggregation would need to revised. Another limitation of our method is underscored by the difficultly of enumerating individuals. Counting the number of fish in a refuge was possible in some instances, but in others the density is simply too high, and making it impossible to discern fish that have already been enumerated from ones that have not. Finally, the seemingly sample size (*n* = 12) in this study is a source of uncertainty. Whilst the sample size is small, it must be viewed in relation to the phenomenon it represents. These fish have high thermal tolerances, with the first aggregation occurring at 27°C (also see Corey, 2022). It is not uncommon for the main-stem of the LSW-M to reach temperatures between 25°C and 30°C during the summer (Morgan and O’Sullivan, 2022), the mean temperatures during this study period were 21.8°C and 20.8°C during 2020 and 2021, respectively. As such, the number of days throughout a summer when river temperatures reach the threshold to initiate behavioural thermoregulation in juveniles is relatively low. Even so, our models were transferable between years, thereby offering credence to the conceptual model and the underlying mechanism.

## Data availability statement

The underlying data for the analyses performed in this study is included as an.xlsl file in the supplementary information.

## Authors’ contribution

**AM.O’S**: conceptualized and designed the study, built camera systems, conducted field work, developed analytical models and wrote the first draft; **E.M.C.**: reviewed and edited; **E.N.C.**: reviewed and edited; **J.H.**: built camera systems, reviewed and edited; **R.A.C.**: reviewed and edited; **C. M.**: built camera systems, reviewed and edited; **T.L.**: conceptualized and designed the study, reviewed and edited.

## References

[ref1] Andrews SN , O’Sullivan AM, Helminen J, Arluison DF, Samways KM, Linnansaari T, Curry RA (2020) Development of active numerating side-scan for a high-density overwintering location for endemic shortnose sturgeon (Acipenser brevirostrum) in the Saint John River, New Brunswick. Diversity 12. 10.3390/d12010023.

[ref2] Brassard P , Ferland-Dutil H, Smirl JD, Paquette M, Le Blanc O, Malenfant S, Ainslie PN (2017) Evidence for hysteresis in the cerebral pressure-flow relationship in healthy men. American Journal of Physiology - Heart and Circulatory Physiology 312: H701–H704. 10.1152/ajpheart.00790.2016. 28130339

[ref3] Breau C , Cunjak RA, Bremset G (2007) Age-specific aggregation of wild juvenile Atlantic salmon Salmo salar at cool water sources during high temperature events. J Fish Biol 71: 1179–1191. 10.1111/j.1095-8649.2007.01591.x.

[ref4] Breau C , Cunjak RA, Peake SJ (2011) Behaviour during elevated water temperatures: can physiology explain movement of juvenile Atlantic salmon to cool water? Journal of Animal Ecology 80: 844–853. 10.1111/j.1365-2656.2011.01828.x. 21401593

[ref5] Brewitt KS , Danner EM (2014) Spatio-temporal temperature variation influences juvenile steelhead (Oncorhynchus mykiss) use of thermal refuges. Ecosphere 5: art92. 10.1890/ES14-00036.1.

[ref6] Brewitt KS , Danner EM, Moore JW (2017) Hot eats and cool creeks: juvenile Pacific salmonids use mainstem prey while in thermal refuges. Can. J. Fish. Aquat. Sci 74: 1588–1602. 10.1139/CJFAS-2016-0395.

[ref7] Brodeur NN , Hébert C, Caissie D, Breau C (2015) Predicting stream temperatures under a climate change scenario: impacts on critical temperatures for Atlantic salmon (Salmo salar). Moncton, NB, Retrieved from http://publications.gc.ca/collections/collection_2015/mpo-dfo/Fs97-6-3118-eng.pdf

[ref8] Brown JH , Gillooly JF, Allen AP, Savage VM, West GB (2004) Toward a metabolic theory of ecology. Ecology 85: 1771–1789. 10.1890/03-9000.

[ref9] Caissie D , Breau C, Hayward J, Cameron P (2013) Water temperature characteristics of the Miramichi and Restigouche River. Moncton, NB, Retrieved from https://dfo-mpo.gc.ca/csas-sccs/publications/resdocs-docrech/2012/2012_165-eng.html

[ref10] Chaput G , Douglas SG, Hayward J (2016) Biological Characteristics and Population Dynamics of Atlantic Salmon (Salmo salar) from the Miramichi River, New Brunswick, Canada. Moncton, NB, Canadian Science Advisory Secretariat (CSAS) Research Document

[ref11] Clarke, A. (2006) Temperature and the metabolic theory of ecology. Functional Ecology 20: 405–412. 10.1111/j.1365-2435.2006.01109.x

[ref12] Corey E (2022) The biological significance of thermal refuges to juvenile Atlantic salmon (Salmo salar) in a changing climate. University of New Brunswick, Fredericton, New Brunswick, Canada.

[ref13] Corey E , Linnansaari T, Cunjak RA, Currie S (2017) Physiological effects of environmentally relevant, multi-day thermal stress on wild juvenile Atlantic salmon (Salmo salar). Conservation Phys Ther 5: cox014. 10.1093/conphys/cox014. PMC5386008 28413684

[ref14] Corey E , Linnansaari T, Dugdale SJ, Bergeron N, Gendron J, Lapointe M, Cunjak RA (2020) Comparing the behavioural thermoregulation response to heat stress by Atlantic salmon parr (*Salmo salar*) in two rivers. Ecology of freshwater fish 29: 50–62. 10.1111/eff.12487.

[ref15] Curry RA , Bernatchez L, Whoriskey F, Audet C (2010) The origins and persistence of anadromy in brook charr. Rev Fish Biol Fish 20: 557–570. 10.1007/s11160-010-9160-z.

[ref16] Dugdale SJ , Franssen J, Corey E, Bergeron NE, Lapointe M, Cunjak RA (2016) Main stem movement of Atlantic salmon parr in response to high river temperature. Ecol Freshw Fish 25: 429–445. 10.1111/eff.12224.

[ref17] Ebersole JL , Liss WJ, Frissell CA (2001) Relationship between stream temperature, thermal refugia and rainbow trout Oncorhynchus mykiss abundance in arid-land streams in the northwestern United States. Ecol Freshw Fish 10: 1–10. 10.1034/j.1600-0633.2001.100101.x.

[ref18] Ebersole JL , Liss WJ, Frissell CA (2003) Cold water patches in warm streams: physicochemical characteristics and the influence of shading. J Am Water Resour Assoc 39: 355–368. 10.1111/j.1752-1688.2003.tb04390.x.

[ref19] Elliott JM (1991) Tolerance and resistance to thermal stress in juvenile Atlantic salmon, Salmo salar. Freshw Biol 25: 61–70. 10.1111/J.1365-2427.1991.TB00473.X.

[ref20] Elliott JM , Elliott JA (2010) Temperature requirements of Atlantic salmon Salmo salar, brown trout Salmo trutta and Arctic charr Salvelinus alpinus: Predicting the effects of climate change. J Fish Biol 77: 1793–1817. 10.1111/j.1095-8649.2010.02762.x. 21078091

[ref21] Fitzgerald AM , Martin BT (2022) Quantification of thermal impacts across freshwater life stages to improve temperature management for anadromous salmonids. Conservation Phys Ther 10. 10.1093/CONPHYS/COAC013. PMC9041423 35492417

[ref22] Frechette DM , Dionne M, Dodson JJ, Bergeron NE (2021) Atlantic salmon movement patterns and habitat use during colonization of novel habitat. Proceedings of the American Fisheries Society 150: 327–344. 10.1002/tafs.10284.

[ref23] Gallant MJ , LeBlanc S, MacCormack TJ, Currie S (2017) Physiological responses to a short-term, environmentally realistic, acute heat stress in Atlantic salmon, Salmo salar. Facets (Ott) 2: 330–341. 10.1139/FACETS-2016-0053.

[ref25] Hermann A , Chladek J, Stepputtis D (2020) iFO (infrared fish observation) – an open source low-cost infrared underwater video system. HardwareX 8: e00149. 10.1016/j.ohx.2020.e00149. 35498235 PMC9041171

[ref26] Hollister RD , Webber PJ, Bay C (2005) Plant response to temperature in northern ALASKA: implications for PREDICTING vegetation change. Ecology 86: 1562–1570. 10.1890/04-0520.

[ref27] Huntsman AG (1942) Death of Salmon and Trout with high temperature. J Fish Res Board Can5c: 485–501. 10.1139/f40-051.

[ref28] Huusko A , Greenberg L, Stickler M, Linnansaari T, Nykänen M, Vehanen T, Alfredsen K (2007) Life in the ice lane: the winter ecology of stream salmonids. River Res Appl 23: 469–491. 10.1002/rra.999.

[ref29] Isaak DJ , Young MK, Nagel DE, Horan DL, Groce MC (2015) The cold-water climate shield: delineating refugia for preserving salmonid fishes through the 21st century. Glob Chang Biol 21: 2540–2553. 10.1111/gcb.12879. 25728937

[ref30] Jiles DC (1994) Frequency dependence of hysteresis curves in conducting magnetic materials. J Appl Phys 76: 5849–5855. 10.1063/1.358399.

[ref31] Keefer ML , Caudill CC (2016) Estimating thermal exposure of adult summer steelhead and fall Chinook salmon migrating in a warm impounded river. Ecol Freshw Fish 25: 599–611. 10.1111/EFF.12238.

[ref32] Kurylyk BL , Bourque CP-A, MacQuarrie KTB (2013) Potential surface temperature and shallow groundwater temperature response to climate change: an example from a small forested catchment in east-Central New Brunswick (Canada). Hydrol Earth Syst Sci 17: 2701–2716. 10.5194/hess-17-2701-2013.

[ref33] Kurylyk BL , MacQuarrie KTB, Caissie D, McKenzie JM (2014) Shallow groundwater thermal sensitivity to climate change and land cover disturbances : derivation of analytical expressions and implications for stream temperature projections. Hydrology and Earth System Sciences Discussions 11: 12573–12626. 10.5194/hessd-11-12573-2014.

[ref34] Lennox RJ , Eliason EJ, Havn TB, Johansen MR, Thorstad EB, Cooke SJ, Uglem I (2018) Bioenergetic consequences of warming rivers to adult Atlantic salmon Salmo salar during their spawning migration. Freshwater Biology 63: 1381–1393. 10.1111/FWB.13166.

[ref35] Lewis JM , Hori TS, Rise ML, Walsh PJ, Currie S (2010) Transcriptome responses to heat stress in the nucleated red blood cells of the rainbow trout (Oncorhynchus mykiss). Physiol Genomics 42: 361–373. 10.1152/physiolgenomics.00067.2010. 20551145 10.1152/physiolgenomics.00067.2010

[ref36] Linnansaari T , Cunjak RA (2010) Patterns in apparent survival of Atlantic salmon (Salmo salar) parr in relation to variable ice conditions throughout winter. Can J Fish Aquat Sci 67: 1744–1754. 10.1139/F10-093.

[ref37] Little AG , Loughland I, Seebacher F (2020) What do warming waters mean for fish physiology and fisheries? J Fish Biol 97: 328–340. 10.1111/jfb.14402. 32441327

[ref38] Lund SG , Caissie D, Cunjak RA, Vijayan MM, Tufts BL (2002) The effects of environmental heat stress on heat-shock mRNA and protein expression in Miramichi Atlantic salmon (Salmo salar) parr. Can J Fish Aquat Sci 59: 1553–1562. 10.1139/f02-117.

[ref39] Lund SG , Lund MEA, Tufts BL (2003) Red blood cell Hsp 70 mRNA and protein as bio-indicators of temperature stress in the brook trout (*Salvelinus fontinalis*). Can J Fish Aquat Sci 60: 460–470. 10.1139/F03-039.

[ref40] Martin BT , Dudley PN, Kashef NS, Stafford DM, Reeder WJ, Tonina D, Danner EM (2020) The biophysical basis of thermal tolerance in fish eggs. Proc R Soc B 287: 20201550. 10.1098/RSPB.2020.1550. PMC7661308 33081621

[ref41] Mesa MG , Weiland LK, Wanger P (2002) Effects of acute thermal stress on the survival, predator avoidance, and physiology of juvenile fall Chinook salmon. Northwest Science 76: 1818–1128 Retrieved from https://pubs.er.usgs.gov/publication/70170575.

[ref42] Morash AJ , Speers-Roesch B, Andrew S, Currie S (2021) The physiological ups and downs of thermal variability in temperate freshwater ecosystems. J Fish Biol 98: 1524–1535. 10.1111/jfb.14655. 33349944

[ref43] Morelli TL , Barrows CW, Ramirez AR, Cartwright JM, Ackerly DD, Eaves TD, Thorne JH (2020) Climate-change refugia: biodiversity in the slow lane. Front Ecol Environ 18: 228–234. 10.1002/fee.2189. 33424494 PMC7787983

[ref44] Morgan AM , O’Sullivan AM (2022) Cooler, bigger; warmer, smaller: fine-scale thermal heterogeneity maps age class and species distribution in behaviourally thermoregulating salmonids. River Res Appl . 10.1002/RRA.4073.

[ref45] O’Sullivan AM , Corey EM, Cunjak RA, Linnansaari T, Curry RA (2021b) Salmonid thermal habitat contraction in a hydrogeologically complex setting. Ecosphere 12: e03797. 10.1002/ECS2.3797.

[ref46] O’Sullivan AM , Linnansaari T, Curry RA (2019) Ice cover exists: a quick method to delineate groundwater inputs in running waters for cold and temperate regions. Hydrol Process 33: 3297–3309. 10.1002/hyp.13557.

[ref47] O’Sullivan AM , Linnansaari T, Leavitt J, Samways KM, Kurylyk BL, Curry RA (2021a) The salmon-peloton: hydraulic habitat shifts of adult Atlantic salmon (Salmo salar) due to behavioural thermoregulation. River Res Appl 38: 107–118. 10.1002/RRA.3872.

[ref48] Parkhurst AM (2010) Model for understanding thermal hysteresis during heat stress: a matter of direction. Int J Biometeorol 54: 637–645. 10.1007/s00484-009-0299-z. 20140629

[ref49] Samways KM (2017) The Importance of Marine-derived Nutrients from Anadromous Fishes to Atlantic Rivers. UNB, Fredericton, NB, Retrieved from https://unbscholar.lib.unb.ca/islandora/object/unbscholar%3A9070/

[ref50] Santos M , Castañeda LE, Rezende EL (2011) Making sense of heat tolerance estimates in ectotherms: lessons from drosophila. Functional Ecology 25: 1169–1180. 10.1111/J.1365-2435.2011.01908.X.

[ref51] Shapley H , Ysiolog PH, Shaple Y-H (1924) Note on the Thermokinetics of Dolichoderine ants. Proc Natl Acad Sci 10: 436–439. 10.1073/PNAS.10.10.436. 16576855 PMC1085745

[ref52] Sutton RJ , Deas ML, Tanaka SK, Soto T, Corum RA (2007) Salmonid observations at a Klamath River thermal refuge under various hydrological and meteorological conditions. River Res Appl 23: 775–785. 10.1002/RRA.1026.

[ref53] Torgersen CE , Price DM, Li HW, McIntosh BA (1999) Multiscale thermal refugia and stream habitat associations of Chinook salmon in northeastern Oregon. Ecol Appl 9: 301–319. 10.1890/1051-0761(1999)009[0301:MTRASH]2.0.CO;2.

[ref54] Tunnah L , Currie S, MacCormack TJ (2017) Do prior diel thermal cycles influence the physiological response of Atlantic salmon (Salmo salar) to subsequent heat stress? Can J Fish Aquat Sci 74: 127–139. 10.1139/cjfas-2016-0157.

[ref55] Wilbur NM , O’Sullivan AM, MacQuarrie KTB, Linnansaari T, Curry RA (2020) Characterizing physical habitat preferences and thermal refuge occupancy of brook trout (*Salvelinus fontinalis*) and Atlantic salmon (*Salmo salar*) at high river temperatures. River Res Appl 36: 769–783. 10.1002/rra.3570.

[ref56] Wondzell SM , Ward AS (2022) The channel-source hypothesis: empirical evidence for in-channel sourcing of dissolved organic carbon to explain hysteresis in a headwater mountain stream. Hydrol Process 36: e14570. 10.1002/HYP.14570.

